# Women and Autoimmune Diseases^1^

**DOI:** 10.3201/eid1011.040367

**Published:** 2004-11

**Authors:** DeLisa Fairweather, Noel R. Rose

**Affiliations:** * Johns Hopkins University, Baltimore, Maryland, USA

**Keywords:** autoimmunity, myocarditis, cytokines, innate immunity, virus hormones, conference report

## Abstract

Autoimmune diseases affect approximately 8% of the population, 78% of whom are women. The reasons for the high prevalence in women are unknown, but circumstantial evidence links autoimmune diseases with preceding infections. Animal models of autoimmune diseases have shown that infections can induce autoimmune disease. For example, coxsackievirus B3 (CB3) infection of susceptible mice results in inflammation of the heart (myocarditis) that resembles myocarditis in humans. The same disease can be induced by injecting mice with heart proteins mixed with adjuvant(s), which indicates that an active infection is not necessary for the development of autoimmune disease. We have found that CB3 triggers autoimmune disease in susceptible mice by stimulating elevated levels of proinflammatory cytokines from mast cells during the innate immune response. Sex hormones may further amplify this hyperimmune response to infection in susceptible persons, which leads to an increased prevalence of autoimmune diseases in women.

## Abstract 2

Recent evidence indicates that sex hormones may exacerbate autoimmune diseases, particularly in women, by increasing the adjuvant effect of infections.

Autoimmune diseases are the third most common category of disease in the United States after cancer and heart disease; they affect approximately 5%–8% of the population or 14–22 million persons (*1*). Autoimmune diseases can affect virtually every site in the body, including the endocrine system, connective tissue, gastrointestinal tract, heart, skin, and kidneys. At least 15 diseases are known to be the direct result of an autoimmune response, while circumstantial evidence implicates >80 conditions with autoimmunity (*2*). In several instances, such as rheumatoid arthritis, multiple sclerosis, and myocarditis, the autoimmune disease can be induced experimentally by administering self-antigen in the presence of adjuvant (collagen, myelin basic protein, and cardiac myosin, respectively) (*3*). An important unifying theme in autoimmune diseases is a high prevalence in women (Figure 1) (*4**,**5*). Conservative estimates indicate that 6.7 million or 78.8% of the persons with autoimmune diseases are women (*4*).

**Figure 1:**
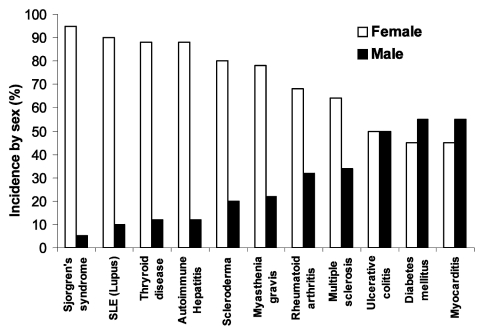
Major autoimmune diseases, comparing the incidence of disease in women (white bar) to the incidence in men (black bar) by percentage. Modified from (*5*).

Soon after autoimmune diseases were first recognized more than a century ago, researchers began to associate them with viral and bacterial infections. Autoimmune diseases tend to cluster in families and in individuals (a person with one autoimmune disease is more likely to get another), which indicates that common mechanisms are involved in disease susceptibility. Studies of the prevalence of autoimmune disease in monozygotic twins show that genetic as well as environmental factors (such as infection) are necessary for the disease to develop (*6*). Genetic factors are important in the development of autoimmune disease, since such diseases develop in certain strains of mice (e.g., systemic lupus erythematosus or lupus in MRL mice) without any apparent infectious environmental trigger. However, a body of circumstantial evidence links diabetes, multiple sclerosis, myocarditis, and many other autoimmune diseases with preceding infections (Table) (*7**,**8*). More often, many different microorganisms have been associated with a single autoimmune disease, which indicates that more than one infectious agent can induce the same disease through similar mechanisms (Table) (*9*). Since infections generally occur well before the onset of symptoms of autoimmune disease, clinically linking a specific causative agent to a particular autoimmune disease is difficult (Figure 2). This difficulty raises the question of whether autoimmune diseases really can be attributed to infections.

**Table:** Infections in humans associated with autoimmune diseases

Disease	Infection
Multiple sclerosis	Epstein-Barr virus (EBV), measles virus
Lyme arthritis	*Borrelia burgdorferi*
Type I diabetes	Coxsackie virus B4, rubella virus, cytomegalovirus (CMV), mumps virus
Rheumatoid arthritis	*Escherichia coli*, mycobacteria, EBV, hepatitis C virus (HCV)
Lupus erythematosis	EBV
Myocarditis	CB3, CMV, chlamydia
Rheumatic fever/myocarditis	Streptococci
Chagas' disease/myocarditis	*Trypanosoma cruzi*
Myasthenia gravis	Herpes simplex virus, HCV
Guillain-Barré syndrome	CMV, EBV, *Campylobacter* spp.

**Figure 2:**
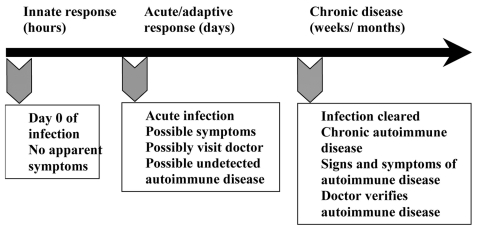
nfections occur before the onset of symptoms of autoimmune disease, making links to specific causative agents difficult. When a person is first infected (day 0), usually no symptoms are apparent. Signs and symptoms of autoimmune disease are clearly present and easily confirmed by physicians during the chronic stage of autoimmunity. However, the infection has been cleared by this time, making it difficult to establish that an infection caused the autoimmune disease. Modified from (*16*).

## Are Autoimmune Diseases Caused by Infections?

To address the question of whether autoimmune diseases can be induced by infections, first autoimmunity needs to be defined. Autoimmune disease occurs when a response against a self-antigen(s) involving T cells, B cells, or autoantibodies induces injury systemically or against a particular organ. Understanding of autoimmune diseases is hindered by the fact that some level of autoimmunity, in the form of naturally occurring autoantibodies and self-reactive T and B cells, is present in all normal persons (*6*). Thus, on a proportional basis, developing autoimmune disease is the relatively uncommon consequence of a common autoimmune response. Although an autoimmune response occurs in most persons, clinically relevant autoimmune disease develops only in susceptible persons (Figure 2).

Given those circumstances, how can infections induce autoimmune disease? A mechanism often called on to explain the association of infection with autoimmune disease is "molecular mimicry," that is, antigens (or more properly epitopes) of the microorganism closely resemble self-antigens. The induction of an immune response to the microbial antigen thus results in cross-reaction with self-antigens and induction of autoimmunity (*10*). Although epitope-specific cross-reactivity between microbes and self-tissues has been shown in some animal models (*11**,**12*), molecular mimicry has not been clearly demonstrated to occur in human diseases (*13*). Another possibility is that microorganisms expose self-antigens to the immune system by directly damaging tissues during an active infection. This mechanism has been referred to as the "bystander effect" (*14**,**15*). However, whether pathogens mimic self-antigens, release sequestered self-antigens, or both, is difficult to determine.

In addition to antigen-specific mechanisms, nonspecific mechanisms could also lead to autoimmunity after infection (*9**,**16*). Activation of the innate immune system is essential for a protective adaptive immune response to develop; and vaccines that lack intrinsic activation of innate immunity (e.g., subunit vaccines) require microbial adjuvants to be immunogenic (*17*). Historically, adjuvants are considered to stimulate immune responses nonspecifically. A renewed understanding of the critical role of innate immunity in influencing the development of an adaptive immune response has led researchers to a better understanding of "the adjuvant effect" (*16*). Although innate immune cells do not respond to specific antigenic epitopes on pathogens, they do produce restricted responses to particular classes of pathogens through pattern-recognition receptors (PRR), such as Toll-like receptors (TLR) (*18*). Interaction of the microorganism component of adjuvants with PRR on innate immune cells results in activation of antigen-presenting cells and upregulation of molecules essential for antigen presentation, such as major histocompatibility complex (MHC) class II and B7–1/2, as well as production of proinflammatory cytokines. This activation of PRR by the microbial components of adjuvants stimulates the immune response in a manner similar to pathogens such as bacteria or viruses (*16**,**18*). The pathogen-specific innate immune response is not the same as the nonspecific activation that occurs after mechanical tissue damage, such as during surgery. During mechanical injury, self-antigens and cytokines are released without consistently stimulating pathogen-specific responses. Autoimmune disease rarely develops and usually resolves spontaneously, as seen in postcommissurotomy syndrome (or postcardiotomy syndrome). Adjuvants (usually bacterial, e.g., *Mycobacterium* in complete Freund's adjuvant) activate the innate immune response in the same pathogen-specific manner when administered with self-antigen; this process results in organ-specific autoimmune disease in animal models (*9**,**16*). Adjuvant alone (without self-antigen) does not usually result in autoimmune disease, and microorganisms likely provide not only the adjuvant effect to stimulate the immune response but also the damage necessary to make self-antigens available to the immune system, resulting in autoimmune disease.

To determine whether infection can lead to autoimmune disease, direct evidence (e.g., the ability to transfer autoimmune disease), indirect evidence (e.g., the ability to reproduce autoimmune disease in animal models), and circumstantial evidence (e.g., the association of autoantibodies with disease in appropriate clinical settings) should be considered (*3**,**6*). The best evidence so far that infections can induce autoimmune diseases comes from animal models. In most animal models of autoimmunity, including myocarditis, disease has been transferred to naïve animals with autoimmune cells (splenocytes or T cells), autoantibodies (*7*), or both, which provides compelling evidence that infections induce autoimmune diseases by immune-mediated mechanisms.

## Lessons from Coxsackievirus B3 (CB3) Myocarditis

### Genetics of Susceptibility to Myocarditis

Genetic background accounts for only about one third (30%–35%) of the risk of autoimmune disease (*6**,**19*). This estimate is based on studies that compared genetically identical, monozygotic twins to nonidentical, dizygotic pairs, for which the concurrence rate can be as low as 2% to 7% (*19*). Therefore, noninherited factors account for the remaining (approximately 70%) risk of developing an autoimmune disorder. Yet, even identical twins do not have identical immune systems. Genes outside of the MHC contribute to the risk for autoimmune disease. However, little information is available about the function of these non-MHC genes. Recent studies have focused on regulatory signals, and considerable evidence exists that cytotoxic lymphocyte antigen–4 (CTLA-4), which provides a downregulatory signal, influences susceptibility to autoimmunity (*20*). Genes that involve apoptosis, a common pathway by which immune responses generally are terminated, may also predispose persons to autoimmune disease (*6*).

To better understand the relationship between infection and autoimmune disease, we established a mouse model of myocarditis, or inflammation of the heart, induced by CB3 infection (*9*). CB3 is believed to account for most cases of myocarditis in North America and Europe; myocarditis also leads to dilated cardiomyopathy, which can result in heart failure and the need for a heart transplant (*21*). The same strain of CB3 that induces myocarditis in humans also induces disease in mice, which makes it an ideal pathogen to study. We found that susceptibility to myocarditis is due primarily to genes that are not part of the MHC (*22*). Our initial investigations into the genetic predisposition of autoimmune myocarditis involved infecting many inbred mouse strains with CB3 (*22*). Genetic analysis comparing susceptibility loci between susceptible and resistant strains of mice found that susceptibility to myocarditis is associated with genes on mouse chromosomes 1 and 6 that have been previously associated with other autoimmune diseases in mice and humans (M. Guler and N.R. Rose, unpub. data). In susceptible strains of mice ( e.g., BALB/c, A/J), acute heart inflammation develops approximately 7–12 days after infection; the inflammation resolves by day 21, and then a chronic phase of inflammation and dilated cardiomyopathy develops from day 35 (*9*). In contrast, only the acute phase of the disease develops in resistant strains like C57BL/6. After mice are infected with CB3, autoantibodies are produced against cardiac myosin, the major component of heart muscle. We found that susceptible strains of mice produce higher titers of immunoglobulin (Ig) G autoantibodies that are specific for cardiac, but not skeletal, myosin, with an IgG1 response (T-helper 2 [Th2])–type) being prominent (*9*). The predominant cellular infiltrate during the acute phase of CB3-induced myocarditis includes macrophages, neutrophils, CD4+ T cells, and CD8^+^ T cells (S. Frisancho-Kiss et al, unpub. data). Smaller numbers of natural killer cells, B cells, and eosinophils are also present. Both T-cell–mediated and autoantibody-mediated mechanisms have been shown to be important in the development of CB3-induced heart disease in BALB/c mice (*9*).

Knowing that cardiac myosin/adjuvant immunization induces myocarditis similar to CB3 infection, we examined the myosin sequences responsible for disease induction. We found that none of the cardiac myosin sequences were cross-reactive with viral sequences (*23*). Furthermore, cross-reactivity between antibodies induced by myosin immunization or CB3 infection was not observed, which suggests that molecular mimicry is not a predominant mechanism in the development of CB3-induced myocarditis (*13**,**15**,**23*). Viral infections can induce damage to host tissues by direct (e.g., viral replication) or indirect (e.g., nitric oxide) mechanisms. In our model of CB3 myocarditis, however, we did not observe damage to the heart cells during the acute phase of disease (*9**,**24*). We found that CB3 replicates at a relatively low level in the heart and that necrosis and fibrosis did not appear until the chronic phase of disease, after virus had been cleared from the heart (*25*). Thus, a low level of viral replication is sufficient to provide cardiac myosin to the immune system. Overall, our studies of CB3-induced myocarditis favor the hypothesis that autoimmune disease is induced after viral infection of susceptible mice because the pathogen facilitates the release of cardiac myosin and nonspecifically stimulates the innate immune response in a manner similar to the effect of adjuvants (*16*).

### Is Virus Associated with Myocarditis?

Many different microorganisms (e.g., streptococci, *Trypanosoma*, cytomegalovirus [CMV], and CB3) have been associated with the same autoimmune disease (e.g., myocarditis) (Table). We have shown that two completely different viruses (CB3, a small nonenveloped RNA virus, and murine CMV, a large enveloped DNA virus) induce a similar biphasic myocarditis in susceptible BALB/c mice (*9*). Although infectious CB3 or murine CMV (MCMV) can be detected during the acute phase of myocarditis, viral levels do not correlate with the severity of inflammation (*9**,**26**,**27*). Because viral genome can be detected after infectious virus has been cleared from the heart, latent virus may attract inflammation during the chronic stage of disease. However, when we examined the heart for the presence of latent MCMV, we found that viral genome and transcript were present in mice both susceptible to and resistant to the development of chronic disease (*27*). These results indicate that persistence of virus alone is not the determining factor in the development of chronic myocarditis. Yet the best evidence that active viral infection is not required for myocarditis to develop comes from the demonstration that injecting susceptible mice with cardiac myosin emulsified in adjuvant induces experimental autoimmune myocarditis (*24*). In fact, the pathogenesis of experimental autoimmune myocarditis closely resembles the biphasic myocarditis associated with CB3 or MCMV infection. This finding indicates that the adjuvant effect produced by infections or adjuvants during the innate immune response can lead to the development of autoimmune disease when self-antigen is present. We have found in preliminary studies that the same pattern of TLR expression is induced by CB3 and the *Mycobacterium* in complete Freund's adjuvant, which suggests a common mechanism of activation (*16*).

### Proinflammatory Cytokines Determine the Development of Myocarditis

**Figure 3:**
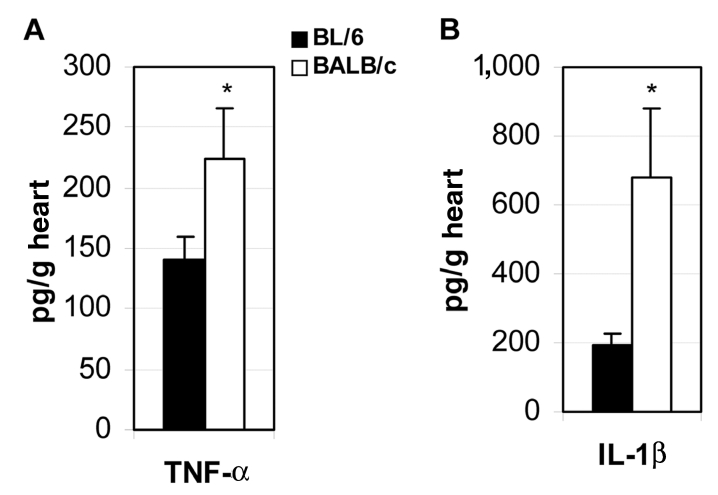
Proinflammatory cytokines are increased in the hearts of susceptible mice during acute myocarditis. Susceptible BALB/c mice were compared to resistant C57BL/6 mice for the level of cytokines tumor necrosis factor (TNF)-α (A) and interleukin (IL)-1β (B) in heart homogenates 12 days after CB3 infection. Data are represented as the mean ± standard error of the mean. *p < 0.05.

Key to understanding the control of susceptibility to autoimmune myocarditis was the finding that adding bacterial lipopolysaccharide (LPS), interleukin (IL)-1β, or tumor necrosis factor (TNF)-α during the innate response to CB3 infection results in the development of the chronic phase of disease in resistant strains of mice (*28**,**29*). Thus, by increasing proinflammatory cytokine production during the innate immune response to infection, genetic resistance to the development of autoimmune disease can be altered. We have found that susceptible BALB/c mice have significantly increased levels of the proinflammatory cytokines TNF-α (Figure 3A) and IL-1β (Figure 3B) in the heart during acute CB3 myocarditis. In fact, many autoimmune diseases, such as rheumatoid arthritis, are associated with increases in TNF-α and IL-1β levels, and treatments that block these cytokines have proven beneficial in animal models and clinical settings (*30*). We have a long-standing interest in the adjuvant effect of lipopolysaccharide (LPS) on the development of autoimmune disease (*28**,**29**,**31*), but only recently has LPS been shown to mediate its effects in part by increasing TNF-α, IL-1β, and IL-18 levels through TLR4 signaling (*18*). Recently, we demonstrated that CB3 infection increases IL-1β and IL-18 levels in the heart during acute myocarditis through IL-12Rβ1 and TLR4 signaling (*26*). Furthermore, the severity of acute myocarditis directly correlates with increased levels of IL-1β and IL-18 in the heart (*26*). Similarly, in the experimental autoimmune myocarditis model, IL-12Rβ1 signaling and increased IL-1β levels are associated with the development of myocarditis (*24*). This effect of LPS or TNF-α on the development of myocarditis is not limited to CB3 infection, but is also observed following MCMV infection (*32*). Thus, proinflammatory cytokine production is key in determining whether susceptible strains of mice develop autoimmune disease after infection.

### Innate Immune Response Initiates Myocarditis

**Figure 4:**
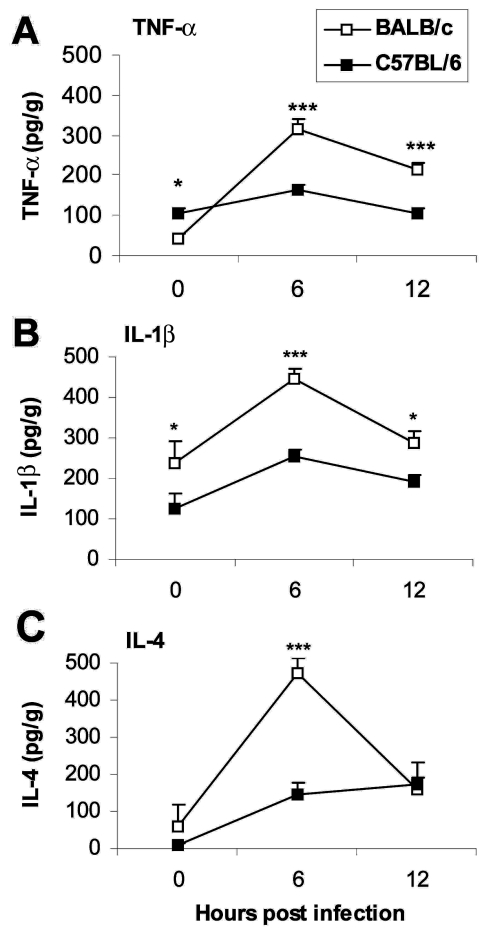
Proinflammatory cytokines are increased in the hearts of susceptible mice during the innate immune response. Susceptible BALB/c mice were compared to resistant C57BL/6 mice for the level tumor necrosis factor (TNF)-α (A), interleukin (IL)-1β (B), and IL-4 (C) cytokines in heart homogenates 6 and 12 hours after CB3 infection. Data are represented as the mean ± standard error of the mean. *p < 0.05; ***p < 0.001. Modified from (*33*).

Since the innate immune response is critical in determining the development of adaptive immunity (*18*) and proinflammatory cytokines administered during the innate response determine whether chronic myocarditis develops, we were interested in studying early differences in the cytokine response to CB3 infection in susceptible (BALB/c) or resistant (C57BL/6) mice to see if they could provide clues to the progression to autoimmunity. We found that susceptible and resistant mice produce the same cytokine profile during the innate immune response to CB3 infection but that susceptible mice have significantly higher levels of cytokines in the heart (Figure 4) and spleen (*33*). The proinflammatory cytokines TNF-α (Figure 4A) and IL-1β (Figure 4B) are significantly increased in susceptible BALB/c mice at 6 and 12 hours after CB3 infection, during the innate immune response. Surprisingly, IL-4 (the prototypic Th2 type cytokine) is also significantly increased 6 hours after CB3 infection (Figure 4C). According to the current dogma, inflammatory autoimmune diseases such as myocarditis are primarily attributable to Th1 responses, with interferon (IFN)-γ as the prototypic cytokine; Th2 responses where IL-4 dominates are believed to reduce autoimmunity. Although protection against viral infections is usually associated with Th1 responses attributable to the protective effect of IFN-γ, in fact, a number of viral infections produce a mixed Th1/Th2 profile, including CB3 (*24**,**25**,**33*). We also observe a mixed Th1/Th2 cytokine profile in the experimental autoimmune myocarditis model (*24**,**34*).

**Figure 5:**
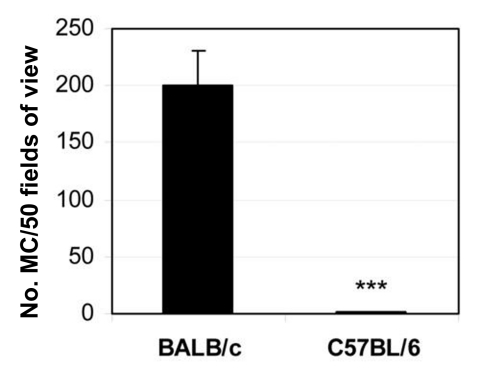
Mast cells are increased in the spleens of susceptible BALB/c mice 6 hours after CB3 infection. Data are represented as the mean ± standard error of the mean. ***p < 0.001. Modified from (*33*).

An elevated IL-4, TNF-α, and IL-1β response is reminiscent of the hypersensitivity reaction of mast cells during allergic responses (*35*). Mast cells are known to produce a rapid burst of cytokines (e.g., TNF-α, IL-1β, and IL-4) when stimulated through TLRs such as TLR2 and TLR4 (*36*). When we looked for mast cells in the spleen 6 hours after CB3 infection, we found that susceptible BALB/c mice had significantly more mast cells than resistant C57BL/6 mice (Figure 5) (*33*). We also found that TLR4 is increased on mast cells of susceptible mice immediately after infection (*16*). Thus, the increased innate cytokine response to CB3 in susceptible mice may be due to a mast cell–mediated response to pattern recognition sequences on CB3 (*16**,**18**,**33*), similar to an allergic reaction. Since the innate immune response determines whether the chronic phase of myocarditis and dilated cardiomyopathy develop in mice (*29**,**32*), early activation of mast cells may result in a delayed-type hypersensitivity reaction later, during the chronic phase of the disease (*25*). Mast cells are found in the human heart in increased numbers during cardiovascular disease and congestive heart failure (*37*). We have also observed increased numbers of degranulating mast cells during chronic CB3 myocarditis in susceptible mice with severe disease (*25*). So the evidence presented by the CB3-induced model of myocarditis demonstrates that virus can trigger autoimmune disease in susceptible mice by immune-mediated mechanisms. But the question still remains: Why are autoimmune diseases so prevalent in women?

## Why Are Autoimmune Diseases So Prevalent in Women?

Even though women's greater susceptibility to autoimmune diseases has been recognized for more than 100 years, only recently has attention focused on this topic (*5*). For some time, the basic immune response between men and women has been known to differ, with women producing a more vigorous immune response and increased antibody production (*5**,**38*). However, autoimmune diseases that develop in men often are more severe (*39*). Most of our understanding of sex differences in the immune response comes from work done in animal models. Many animal models of autoimmune disease have shown a similar sex bias, with a higher incidence of disease in women. Sex hormones, such as estrogen, testosterone, and progesterone, may mediate most of the sex-biased differences in the immune response (*39*). Recently, estrogens and androgens have been found to directly influence whether a Th1- or Th2-type immune response develops by interacting with hormone receptors on immune cells (*38*). Not only are a variety of sex hormone receptors found on immune cells, but cytokine receptors (e.g., IL-1R, IL-18R) have likewise been discovered on hormone-producing tissues, which suggests bidirectional regulation of the immune response. Furthermore, proinflammatory cytokines such as TNF-α and IL-1β stimulate the release of glucocorticoids from the hypothalamus-pituitary-adrenal axis, which regulates the inflammatory process, along with androgens and estrogens (*40*).

**Figure 6:**
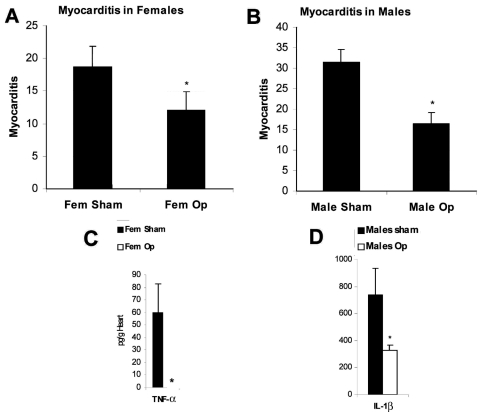
Sex hormones increase myocarditis in female and male mice by increasing interleukin (IL)-1β and tumor necrosis factor (TNF)-α levels in the heart. Susceptible female (A,C) and male (B,D) BALB/c mice underwent gonadectomy (Fem op/Male op) and were compared to sham-operated controls (Fem sham/Male sham) for the level of myocarditis (% inflammation) and cytokines (pg/g) in the heart after CB3 infection. CB3 myocarditis was assessed for (A) female mice and (B) male mice after the operation. Data are represented as the mean ± standard error of the mean. *p < 0.05.

The precise interaction between hormones and the innate immune response after infection is poorly understood. However, in vitro studies of immune cells cultured in the presence of hormones have shown that estrogen significantly increases proinflammatory cytokine production (e.g., TNF-α, IL-1β) (*5*). In preliminary experiments studying the role of sex hormones on the development of CB3-induced myocarditis in mice, we have found that sex hormones increase inflammation and proinflammatory cytokines in the hearts of male and female mice after infection. Gonadectomy before CB3 infection reduces myocarditis in female (Figure 6A) and male mice (Figure 6B). Reduced inflammation is associated with reduced TNF-α in the female heart (Figure 6C) and reduced IL-1β in the male heart (Figure 6D). Thus, the elevated immune response in women may even further amplify the adjuvant effect of infection, thereby increasing the possibility that chronic, autoimmune disease will subsequently develop in women. With the increase in the number of autoimmune cases in recent years, the possible role of infections in exacerbating disease, particularly in women, is of rising concern.
